# The Law of Lunacy
*"A Letter to the Right Hon. Lord Ashley, M.P., relative to the Case of Nottidge *versus* Ripley. By T. T. Wingett, M.D., Medical Superintendent of the Dundee Royal Lunatic Asylum. Dundee. 1849."—2. "The Defects of the Law of Lunacy: a Vindication of the Decision of the Lord Chief Baron, in the Case of Nottidge *verses* Ripley. By T. T. Wingett, M.D. Dundee. 1849."


**Published:** 1850-01-01

**Authors:** 


### Art. II.—THE LAW OF LUNACY.*

These pamphlets refer to the important subject which we discussed at some length in the last number of the Journal. As we have so fully expressed *our* opinion of the dictum of Lord Chief Baron Pollock in the celebrated case of Nottidge *v.* Ripley, it will be unnecessary to enter at any length into the consideration of the question at issue, between the learned judge and that part of the profession qualified, by education, habit of thought, and practical experience, to give a sound and satisfactory opinion in relation to the matter. Dr. Wingett, the author of the two pamphlets before us, whilst he condemns what he designates as the "obnoxious influence" of the Lord Chief Baron's opinion—which he (Dr. Wingett) declares to have "already done much mischief in the judicial proceedings of Scotland" —yet declares that—

"It is, palpably, the grossest absurdity to impeach the judge for the results of this case; he has merely propounded the law—extreme law it may be—but the law nevertheless. The law only is at fault, and if we insist upon attacking the law in the person of his lordship, hold him responsible for all its fatuities, and treat him as the proxy of Sir Edward Coke and his predecessors, Sir Frederick Pollock will stand forth to us in a somewhat similar spirit and attitude to that maintained by Cato, who when, at the age of eighty-six, he was accused of certain offences of his past life, committed at times long gone by, said, with profound sagacity: 'It is difficult to render an account of one's own conduct to men belonging to an age different from that in which one has lived.' "

Again our author observes that—

"Sir Frederick Pollock was obviously led astray in his decision

* "A Letter to the Right Hon. Lord Ashley, M.P., relative to the Case of Nottidge *versus* Ripley. By T. T. Wingett, M.D., Medical Superintendent of the Dundee Royal Lunatic Asylum. Dundee. 1849."—2. "The Defects of the Law of Lunacy: a Vindication of the Decision of the Lord Chief Baron, in the Case of Nottidge *verses* Ripley. By T. T. Wingett, M.D. Dundee. 1849."

by modern writers upon the jurisprudence of insanity, whom he had adopted for his teachers, and who now, forsooth, censure him for giving their tuitions a practical application. Every one acquainted with the modern literature of this country upon mental disease, must know that the onus of this unfortunate dictum does not rest with the Chief Baron. It is to be found in the standard works of our medical libraries. The heresy has a medical, not a legal origin. Its author was a doctor, not a judge. In common fairness, therefore, it must be conceded to Sir Frederick Pollock that he has been misled by a medical opinion—to be found in books, but happily not carried into practice. His excuse is really to be found in his having acted upon the wise apothegm, 'ne sutor ultra crepidem.' "

Dr. Wingett singles out Dr. Conolly and Dr. Wigan as the culprits, and arraigns these two physicians as the real authors of the judgment which has exercised such "obnoxious influence" on public opinion. Dr. Conolly is of age, and can speak for himself. Dr. Wigan, our much respected friend, is sleeping with his fathers; and, therefore, must depend upon those to defend him who had the honour of his friendship, and who had opportunities of becoming acquainted with his opinions. In justice to Dr. Wingett, we admit that the passage he cites from the "Duality of the Mind," somewhat warrants the deduction drawn. The part quoted is as follows—

"For more than forty years I have been familiar with the manifestations of disordered intellect. The moral object which Dr. Conolly seems to have had in view in his 'Inquiry concerning the Indications of Insanity,' was, to exempt from the tender mercies of the law, persons who, although eccentric in their habits, and incapable of exercising all the mental faculties in due order and subordination, were yet possessed of sufficient self-control to *avoid injuring others or themselves,* which *he very justly considers* to be the boundary of the right of interference on the part of society."

We feel, however, convinced that Dr. Wigan did not, in this passage, refer to persons palpably insane, and whose "eccentricity," and incapability of exercising "all the mental faculties in due order and subordination," were the *results of disease.* That much eccentricity and irregularity of thought and action *may exist apart from all disease of mind,* is an indisputable fact. Great eccentricity, mental irregularity, extraordinary conduct, may all be the *natural* operations of the mind. There is healthy "eccentricity," and normal "irregularity," for the cure of which it would be the height of absurdity and cruelty to confine a person. As there exists certain physical deviations from Hogarth's celebrated line of beauty, so there are mental departures from our *à priori* ideas of a sound, and a well-balanced mind, which, instead of being indications of derangement, are to be viewed as symptons of health and sanity! We once heard a distinguished physician who was giving evidence before a commission in lunacy, admit, in answer to a question from a clever barrister, that a man was under a delusion who believed in clairvoyance, and who thought it possible for a person in a mesmeric state of exaltation, to see through a five inch deal board. We consider that the physician in question did not draw a scientific distinction between what may be termed false conclusions, or notions of belief consequent upon a healthy—it may be an ill-educated and illogically constituted mind—and those *creations of the intellect* the effect of a diseased or disordered condition of the nervous apparatus, through which the faculties of the understanding are manifested. A man may entertain most absurd and preposterous notions, and yet be pathologically and legally sane. The fact of his *belief* in these ideas is no evidence of derangement of mind, or of the existence of lunacy.

What, then, it may be asked, constitutes insanity, and what is the *test* of the propriety and necessity of an interference with free agency? These are important questions. Feeling the *impossibility* of giving a definition of insanity which will comprehend within its grasp all the delicate shades of mental disorder, we shall dismiss the former question without making any attempt at its solution. We do not, however, feel the same hesitation in applying our minds to the consideration of the latter interrogatory. If the mind be clearly *disordered*, using that term in its medical and legitimate signification, we have no hesitation in saying, that a *primâ facie* case exists for confinement or surveillance of some kind or other. We may safely predicate that an *insane* person is both dangerous to himself and others, whatever may be the degree of the mental disturbance. This may appear a "dangerous" doctrine to advocate, but it is nevertheless the TRUTH—and as such it is our duty to enforce it. We do not maintain that *every degree* of disturbed or deranged mind, consequent upon some interference with a normal condition of the cerebral organization, justifies an immediate interference with the liberty of the subject; but until we are enabled, by means of the agents, physical and moral, placed at our disposal, to confine or limit the mental derangement to a certain circumscribed line of demarcation, it would be extremely hazardous for us to declare that *such an amount* of insanity renders a person harmless to himself and others; and *another degree, a tittle more in advance,* constitutes him a dangerous lunatic. What may in common *parlance* be viewed as harmless insanity one day, may, by some trifling cause, become dangerous on the following one. A man is suffering from a delusion, fancying people speak to him in the streets, and that he is the subject of almost general observation. He may have these morbid impressions, and be at large, and his own master, for a period, without injuring himself or others. But a little extra mental excitement, a trifling obstruction to the circulation in the liver, a severe fit of indigestion, may make this harmless lunatic a most furious and dangerous person to society. Another insane person may, in his quiet and "harmless" moments, believe himself to be a king; and when the question is mooted as to the propriety of interfering with the free agency of the monomaniac, the medical man who suggests such an idea is hooted at, as if he were, like Vice—

> ——— " A monster of such hideous mien,
> That to be hated needs but to be seen."

But this imaginary king may have a slight attack of illness. His majesty's secretions may become disordered, his bowels costive, an exposure to an extra degree of mental stimuli may interfere with the healthy cerebral circulation, and this individual may not only fancy that he is a regal personage, but may consider that he has human and Divine authority for removing all obstacles that obstruct his full and unfettered exercise of royal power, and under this *extension* of the derangement, he may attempt the life of the sovereign! Such cases have occurred, and may take place again. We cite this illustration to prove *that it is not in our power to predicate that a person is harmless, whose mind is clearly under the influence of disease!* How absurd it would be, if the lungs, heart, or brain were in a state of inflammation, for the physician to say that the attack, if not immediately dangerous to human life, is not likely to become so! The weight of a hair may make all the difference between dangerous and harmless lunacy. Were medical men, conversant with the morbid phenomena of the human mind in all their delicate and subtle phases, not to enforce such a view of the question, they would be culpable in the highest degree. We maintain that no person *positively deranged in mind*, should be permitted to be at large, without being under *some* degree of surveillance. We do not advocate in every case the duty of *confinement* in an asylum; but of the necessity of surveillance there cannot be the shadow of doubt. Society must be protected against the insane, and the insane not being (in consequence of their disease) responsible for their conduct, must be protected *against themselves!* In carrying out this doctrine, great caution should be exercised in discriminating between natural eccentricity, and healthy deviations from the ordinary standard of thought and conduct, and those alterations in the current of ideas and feelings, clearly and conclusively traceable to disorder of the brain and nervous system.

But to return to Dr. Wingett's pamphlet. The passage which our author quotes from Dr. Conolly's writing, and which he conceives justified the Lord Chief Baron in his exposition of the law, is as follows:—

"The fact of the patient's madness can only be established by certain tests of the manner in which his intellectual faculties are exercised, and these tests are to be found in his appearance, in his dress, in the known physical accompaniments of madness, and in his words and actions. *That* is the medical question. The *next* is a medico-legal question, and turns wholly on the disposition of the patient to *injure* himself or his property, or to *injure* others and their property; *and on the probability of such a disposition, though not manifested, being suddenly developed.* On the first question hangs the medical treatment and superintendence: on the second, restraint, confinement, deprivation of authority, and control over property."

We do not consider this passage warrants Dr. Wingett's conclusion. The words we have placed in italics are sufficiently comprehensive to protect Dr. Conolly against the imputation of inconsistency. There is no doubt that the law of lunacy is defective—perhaps like all *human* enactments—necessarily so; but whether our author's suggestion, if carried out, would relieve us from our difficulties it is not in our power to say. He declares that—

"What we want is a statute remedial of the defects of the common law as applicable to lunacy affairs, granting us a power and authority to treat mental unsoundness according to the principles of common sense, benevolence, and scientific enlightenment. In order to this, it will be necessary to enact *two* things. The *one*—that in questions relative to the detention of individuals in lunatic asylums, the definitions of the common law in regard to unsoundness of mind shall be considered weak, narrow-minded, and inaccurate, and therefore null and inoperative. The condition unsound mind, being understood to mean that condition of *mental* or *moral* obliquity or infirmity, which common sense easily recognises as disease or incapacity, but which neither needs nor admits of any precise definition to distinguish it. The *other*—declaring that individuals may be detained in lunatic asylums, if pronounced upon oath by competent individuals to be of unsound mind, and requiring treatment or protection in a lunatic asylum; and that this shall be lawful both for the dangerous and the harmless insane; for the purposes of cure of the mental disease, as well as for the protection of the patients and the public.

"These two enactments would answer the demands of the present hour. It is vain to object to these propositions, that the process of incarceration is too easy. The process of illegal incarceration is easy only in those cases where perjury shall be perpetrated by a number of professional individuals. If a combination of individuals will bear false witness that I stole a watch, I can be incarcerated in the common jail; but who ever dreams of feeling uneasy under such a possible danger? Nor is the danger of false incarceration in a lunatic asylum one jot more imminent or probable. The two foregoing maxims being laid down, the application of them, and everything else relative to lunacy affairs, may be safely entrusted to the dealings of common sense, common honesty, and common humanity."

In reading this passage, the question naturally occurs to the mind, if psychologists have such difficulty in defining what is insanity, admitting at the same time the impossibility of discovering any infallible means of testing its dangerous or harmless tendencies, how is it possible for the Legislature, with all its accumulated wisdom, satisfactorily to untie the gordian knot? We are much disposed to think that any enactments having reference to the abstract points connected with this most mysterious and often inscrutable disease, will be of little avail in producing harmony of opinion among those whose especial province it is to "administer to the mind diseased," or of reconciling the public to the views propounded by our most eminent British psychologists. Our great remedy is in the general promulgation of sound psychological views connected with the subject of insanity. In proportion as the mind becomes enlightened on these matters, so will error and prejudice hide their diminished heads, and truth exercise its just and legitimate influence over public opinion.

---

